# Lack of population genetic structure and host specificity in the bat fly, *Cyclopodia horsfieldi*, across species of *Pteropus* bats in Southeast Asia

**DOI:** 10.1186/1756-3305-6-231

**Published:** 2013-08-08

**Authors:** Kevin J Olival, Carl W Dick, Nancy B Simmons, Juan Carlos Morales, Don J Melnick, Katharina Dittmar, Susan L Perkins, Peter Daszak, Rob DeSalle

**Affiliations:** 1 EcoHealth Alliance, New York, NY 10001, USA; 2 Sackler Institute for Comparative Genomics, American Museum of Natural History, New York, NY 10024, USA; 3 Department of Ecology, Evolution, and Environmental Biology, Columbia University, New York, NY 10027, USA; 4 Dartment of Biology, Western Kentucky University, Bowling Green KY 42101, USA; 5 Department of Zoology, Field Museum of Natural History, Chicago, IL 60605, USA; 6 Department of Mammalogy, American Museum of Natural History, New York, NY 10024, USA; 7 Global Footprint Network, Oakland, CA 94607, USA; 8 Department of Biological Sciences, State University of New York at Buffalo, Buffalo, NY 14260, USA

**Keywords:** *Bartonella*, Connectivity, Diptera, Flying fox, Ectoparasite, Emerging infectious disease, Gene flow, Nipah virus, Nycteribiidae, Pathogens, Phylogeography

## Abstract

**Background:**

Population-level studies of parasites have the potential to elucidate patterns of host movement and cross-species interactions that are not evident from host genealogy alone. Bat flies are obligate and generally host-specific blood-feeding parasites of bats. Old-World flies in the family Nycteribiidae are entirely wingless and depend on their hosts for long-distance dispersal; their population genetics has been unstudied to date.

**Methods:**

We collected a total of 125 bat flies from three *Pteropus* species (*Pteropus vampyrus, P. hypomelanus*, and *P. lylei*) from eight localities in Malaysia, Cambodia, and Vietnam. We identified specimens morphologically and then sequenced three mitochondrial DNA gene fragments (CoI, CoII, cytB; 1744 basepairs total) from a subset of 45 bat flies. We measured genetic diversity, molecular variance, and population genetic subdivision (F_ST_), and used phylogenetic and haplotype network analyses to quantify parasite genetic structure across host species and localities.

**Results:**

All flies were identified as *Cyclopodia horsfieldi* with the exception of two individuals of *Eucampsipoda sundaica*. Low levels of population genetic structure were detected between populations of *Cyclopodia horsfieldi* from across a wide geographic range (~1000 km), and tests for isolation by distance were rejected. AMOVA results support a lack of geographic and host-specific population structure, with molecular variance primarily partitioned within populations. Pairwise F_ST_ values from flies collected from island populations of *Pteropus hypomelanus* in East and West Peninsular Malaysia supported predictions based on previous studies of host genetic structure.

**Conclusions:**

The lack of population genetic structure and morphological variation observed in *Cyclopodia horsfieldi* is most likely due to frequent contact between flying fox species and subsequent high levels of parasite gene flow. Specifically, we suggest that *Pteropus vampyrus* may facilitate movement of bat flies between the three *Pteropus* species in the region. We demonstrate the utility of parasite genetics as an additional layer of information to measure host movement and interspecific host contact. These approaches may have wide implications for understanding zoonotic, epizootic, and enzootic disease dynamics. Bat flies may play a role as vectors of disease in bats, and their competence as vectors of bacterial and/or viral pathogens is in need of further investigation.

## Background

Intraspecific evolutionary studies of parasites can give insight into vector ecology, and uncover patterns of connectivity in host species populations not inferred by host genealogy alone [1,2]. In some cases, host and parasite population structure may be strongly congruent [3,4]. In other cases, host populations may be panmictic or genetically homogeneous, and parasite population structure can be used as a surrogate to identify cryptic population structure or infer host movement [5-8]. Alternatively, structured host populations may harbor unstructured parasite populations, which may suggest high levels of contact (i.e. sharing of parasites), but not mating, among host populations [9,10]. Congruence between host and parasite population structure will vary depending on the life-history traits and ecology of each species, but is generally expected when parasite species are highly host specific, lack free-living stages in their lifecycle, and when host and parasite both have limited dispersal [1,11]. Intraspecific genetic studies of ectoparasites are relatively uncommon in the literature, but there is a growing interest in using molecular data to elucidate parasite-host species interactions at the population level [11-13].

Bat flies (Diptera: Hippoboscoidea) are highly specialized, blood-feeding ectoparasites of bats. The monophyletic group comprises two families, Nycteribiidae and Streblidae, with the latter comprised of Old and New World clades [14]. Nycteribiid bat flies include 3 subfamilies, 12 genera and 275 described species; the subfamily Cyclopodiinae contains 4 genera and 62 species [15]. Flies in the genus *Cyclopodia* parasitize only bat species in the family Pteropodidae, and global distributions of the two groups closely coincide [16]. *Cyclopodia horsfieldi* occurs along with its primary host, *Pteropus vampyrus*, across Malaysia, Indonesia, and the Philippines and there is a recent record of the parasite from *Megaerops niphanae* (Pteropodidae: Cynopteriniae) in Vietnam [16,17]. Most bat fly species are highly host specific, and historical records of multiple host species are often erroneous and should be interpreted with caution [18,19]. In some cases the recorded geographic range of bat fly species is more limited than the host species range, for example *Cyclopodia greeffi* and its widespread host, *Eidolon helvum* in Africa [16]. Although less common, *Cyclopodia* species may exhibit oligoxeny, parasitizing two or more related host species, e.g. *C. albertisi* found on three *Pteropus spp.* in Australia and the islands of Papua New Guinea [16,20]. Marshall examined 44 species of nycteribiids from Malaysia and the New Hebrides and found 29 were monoxenous, parasitizing only one host species, and 15 were oligoxenous [21]. Mechanisms that should be expected to promote host switching in bat flies include a high degree of spatial overlap of host species at the geographic and habitat scale and mixed species roosting, as bat fly pupation takes place off the host and within the roost structure itself [19,22]. However, an extensive study of bat flies in the Neotropics found that specificity of flies followed the taxonomy of hosts, not their ecological associations or polyspecific roosting habits [23]. From an evolutionary perspective, colonizing multiple host species may be an advantageous strategy as this would increase the effective “habitat” available for parasite species to occupy [2].

Nycteribiid bat flies have highly-derived morphological characteristics including winglessness, dorsoventrally flattened bodies, and heads that fold against the thorax when at rest [15]. Bat flies have a unique reproductive strategy, whereby females develop their eggs internally nourished by “milk” glands, and a single prepupa (3^rd^ instar larva) is deposited on the roost substrate. While very little information on nycteribiid biology is available, for *Eucampsipoda* teneral adults typically emerge within ~20-25 days of deposition to seek a new host and feed, and reach sexual maturity in 5–6 days after emergence [24]. Adult flies will die of starvation within 24 hours after being separated from their host. While most streblid species have wings as adults, nycteribiids are wingless and largely dependent on their hosts for dispersal [25].

Large fruit bats in the genus *Pteropus,* commonly called flying foxes, are host to *Cyclopodia* species. Three species of *Pteropus* -- *P. vampyrus*, *P. lylei*, and *P. hypomelanus* -- are broadly sympatric across most of their geographic ranges in Southeast Asia and from what is known, share similar habitat requirements and dietary preference. The Large Flying Fox, *P. vampyrus,* has a wide geographic range from southern Vietnam, Cambodia, Thailand, Malaysia, Philippines, and through much of Indonesia to East Timor [26]. Lyle’s Flying Fox, *P. lylei*, has a narrower range and is found in southern Vietnam, Cambodia, and Thailand [27]. The Variable Flying Fox, *P. hypomelanus,* has a wide and fragmented distribution throughout the Indo-Australian region where it is primarily found on small off-shore islands, often roosting near the coast [28]. All three of these *Pteropus* species can be differentiated in the field using morphological characters and measurements, including forearm length, body mass, size and shape of the pinna, and fur on the dorsal tibia [29,30]. Molecular data support the grouping of these three *Pteropus* species into two distinct clades; *Pteropus vampyrus* and *P. lylei* are part of the same clade, but are not sister taxa to one another [31].

Previous molecular investigations of bat flies have primarily focused on higher-level systematics [14,32]. To our knowledge only two studies have examined the population genetic structure of bat flies, both investigating *Trichobius major* (Streblidae) parasitizing *Myotis velifer* from the USA (Kansas, Oklahoma, and Texas) using mtDNA [33] and amplified fragment length polymorphism of nuclear DNA [34]. Wilson et al. [33] observed only a single mtDNA haplotype for all *T. major* sampled, and Lack et al. [34] identified nDNA variation, but not corresponding to geographic locality.

Here we characterize the genetic and taxonomic diversity and host specificity of bat flies from three species of Southeast Asian fruit bats in the genus *Pteropus* and examine the population genetic structure of *Cyclopodia horsfieldi* across multiple countries using three mitochondrial DNA markers. We discuss these results with respect to host population structure and gene flow, and disease ecology and transmission.

## Methods

### Specimen collection

**Figure 1:**
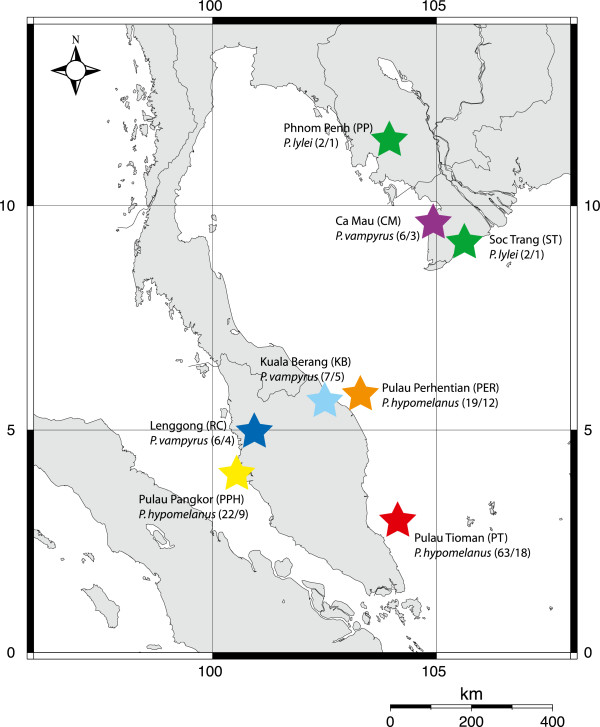
**Sampling localities for *Cyclopodia horsfieldi *(with total flies collected/sequenced) from three *Pteropus *species in Southeast Asia.**

We collected bat flies from three species of flying fox (*Pteropus hypomelanus, P. vampyrus,* and *P. lylei*) from localities across Southeast Asia (Figure 1, Table 1). In Malaysia, canopy mist nets were deployed near diurnal roosting trees or nearby feeding sites, and bats were immediately removed upon capture and held individually in cloth bags for ~1 hr before sampling. Bats were anesthetized using medetomidine/ketamine (0.025/2.5 mg/kg) administered intramuscularly or were restrained without anesthesia to collect ectoparasites and wing biopsies for host DNA [35]. Bat flies were collected with forceps by examining the pelage of anesthetized or restrained bats, and placed directly in 95% ethanol for preservation. All bats were handled and sampled in accordance with IACUC protocol (#AAAA3272) from Columbia University. GPS coordinates were recorded for all sampling localities at the point of capture, although in Vietnam and Cambodia local hunters captured bats alive in mist nets near feeding sites and the exact roost localities were not known but likely within 50 km of the sampling site. All bats were marked, photographed, and released at the site of capture except for individuals of *P. lylei* and *P. vampyrus* from Vietnam which were collected as voucher specimens currently accessioned at the Institute for Ecology and Biological Resources, Hanoi, Vietnam. From July 2004-May 2007, a total of 125 bat flies were collected from 66 individuals of the three host species (Table 1). Fly specimens are currently accessioned at Western Kentucky University, State University of New York at Buffalo, and the American Museum of Natural History. Sampling was not evenly distributed across host species; 104 flies were collected from three populations of *P. hypomelanus*, 17 flies from *P. vampyrus*, and four flies from *P. lylei*.

**Table 1:** Bat fly specimens examined, host species, sampling locations, and genes sequenced

**Bat ID**	**# Flies coll.**	**Fly ID**	**Date collected**	**Fly Morph. ID**	**Host spp.**	**Sampling locality**	**Country**	**GPS Lat (North)**	**GPS Long (East)**	**CytB**	**CoI**	**CoII**
PPH01	2	PPH01	25-Feb-06	*C. horsfieldi*	*P. hypomelanus*	Pulau Pangkor	Malaysia	04.23593°	100.54056°	1	1	1
PPH02	2	PPH02	25-Feb-06	*C. horsfieldi*	*P. hypomelanus*	Pulau Pangkor	Malaysia	04.23593°	100.54056°	1	1	1
PPH03	3	PPH03	25-Feb-06	*C. horsfieldi*	*P. hypomelanus*	Pulau Pangkor	Malaysia	04.23593°	100.54056°	1	1	1
PPH04	2	PPH04	25-Feb-06	*C. horsfieldi*	*P. hypomelanus*	Pulau Pangkor	Malaysia	04.23593°	100.54056°	1	1	1
PPH06	2	PPH06	25-Feb-06	*C. horsfieldi*	*P. hypomelanus*	Pulau Pangkor	Malaysia	04.23593°	100.54056°	1	1	1
PPH08	2	PPH08	26-Feb-06	*C. horsfieldi*	*P. hypomelanus*	Pulau Pangkor	Malaysia	04.23593°	100.54056°	1	1	1
PPH09	4	PPH09	26-Feb-06	*C. horsfieldi*	*P. hypomelanus*	Pulau Pangkor	Malaysia	04.23593°	100.54056°	1	1	1
PPH10	2	PPH10	26-Feb-06	*C. horsfieldi*	*P. hypomelanus*	Pulau Pangkor	Malaysia	04.23593°	100.54056°	1	1	1
PPH12	3	PPH12	26-Feb-06	*C. horsfieldi*	*P. hypomelanus*	Pulau Pangkor	Malaysia	04.23593°	100.54056°	1	1	1
PER1	1	PER01	2-May-07	*C. horsfieldi*	*P. hypomelanus*	Pulau Perhentian	Malaysia	05.90405°	102.74359°	1	1	1
PER2	2	PER02	2-May-07	*C. horsfieldi*	*P. hypomelanus*	Pulau Perhentian	Malaysia	05.90405°	102.74359°	1	--	1
PER3	1	PER03	3-May-07	*C. horsfieldi*	*P. hypomelanus*	Pulau Perhentian	Malaysia	05.90405°	102.74359°	1	1	1
PER6	1	PER06	3-May-07	*C. horsfieldi*	*P. hypomelanus*	Pulau Perhentian	Malaysia	05.90405°	102.74359°	1	1	1
PER7	1	PER07	3-May-07	*C. horsfieldi*	*P. hypomelanus*	Pulau Perhentian	Malaysia	05.90405°	102.74359°	1	1	1
PER8	2	PER08	3-May-07	*C. horsfieldi*	*P. hypomelanus*	Pulau Perhentian	Malaysia	05.90405°	102.74359°	1	1	1
PER9	1	PER09	4-May-07	*C. horsfieldi*	*P. hypomelanus*	Pulau Perhentian	Malaysia	05.90405°	102.74359°	1	1	1
PER10	1	PER10	4-May-07	*C. horsfieldi*	*P. hypomelanus*	Pulau Perhentian	Malaysia	05.90405°	102.74359°	1	1	1
PER11	2	PER11	4-May-07	*C. horsfieldi*	*P. hypomelanus*	Pulau Perhentian	Malaysia	05.90405°	102.74359°	1	1	1
PER12	2	PER12	4-May-07	*C. horsfieldi*	*P. hypomelanus*	Pulau Perhentian	Malaysia	05.90405°	102.74359°	1	1	1
PER13	2	PER13	4-May-07	*C. horsfieldi*	*P. hypomelanus*	Pulau Perhentian	Malaysia	05.90405°	102.74359°	1	1	1
PER14	2	PER14	4-May-07	*C. horsfieldi*	*P. hypomelanus*	Pulau Perhentian	Malaysia	05.90405°	102.74359°	1	1	1
PER15	1	PER15	4-May-07	*C. horsfieldi*	*P. hypomelanus*	Pulau Perhentian	Malaysia	05.90405°	102.74359°	--	--	--
711041	1	PT01	11-Jul-04	*n/a*	*P. hypomelanus*	Pulau Tioman	Malaysia	02.84334°	104.15935°	1	--	--
7110410	2	PT10	11-Jul-04	*C. horsfieldi*	*P. hypomelanus*	Pulau Tioman	Malaysia	02.84334°	104.15935°	1	1	1
7110411	1	PT11	11-Jul-04	*C. horsfieldi*	*P. hypomelanus*	Pulau Tioman	Malaysia	02.84334°	104.15935°	1	1	1
7110412	1	PT12	11-Jul-04	*C. horsfieldi*	*P. hypomelanus*	Pulau Tioman	Malaysia	02.84334°	104.15935°	1	--	--
7110413	1	PT13	11-Jul-04	*C. horsfieldi*	*P. hypomelanus*	Pulau Tioman	Malaysia	02.84334°	104.15935°	1	1	1
711042	3	PT02	11-Jul-04	*C. horsfieldi*	*P. hypomelanus*	Pulau Tioman	Malaysia	02.84334°	104.15935°	--	--	--
711043	1	PT03	11-Jul-04	*n/a*	*P. hypomelanus*	Pulau Tioman	Malaysia	02.84334°	104.15935°	--	--	--
711044	5	PT04	11-Jul-04	*C. horsfieldi*	*P. hypomelanus*	Pulau Tioman	Malaysia	02.84334°	104.15935°	1	1	1
711045	2	PT05	11-Jul-04	*n/a*	*P. hypomelanus*	Pulau Tioman	Malaysia	02.84334°	104.15935°	--	--	--
711046	3	PT06	11-Jul-04	*C. horsfieldi*	*P. hypomelanus*	Pulau Tioman	Malaysia	02.84334°	104.15935°	--	--	--
711047	3	PT07	11-Jul-04	*C. horsfieldi*	*P. hypomelanus*	Pulau Tioman	Malaysia	02.84334°	104.15935°	--	--	--
711048	2	PT08	11-Jul-04	*C. horsfieldi*	*P. hypomelanus*	Pulau Tioman	Malaysia	02.84334°	104.15935°	1	1	1
7120414	2	PT14	12-Jul-04	*C. horsfieldi*	*P. hypomelanus*	Pulau Tioman	Malaysia	02.84334°	104.15935°	--	--	--
7120415	3	PT15	12-Jul-04	*C. horsfieldi*	*P. hypomelanus*	Pulau Tioman	Malaysia	02.84334°	104.15935°	1	1	1
7120416	2	PT16	12-Jul-04	*C. horsfieldi*	*P. hypomelanus*	Pulau Tioman	Malaysia	02.84334°	104.15935°	1	1	1
7120417	2	PT17	12-Jul-04	*C. horsfieldi*	*P. hypomelanus*	Pulau Tioman	Malaysia	02.84334°	104.15935°	--	--	--
7120418	2	PT18	12-Jul-04	*C. horsfieldi*	*P. hypomelanus*	Pulau Tioman	Malaysia	02.84334°	104.15935°	1	1	1
7120419	2	PT19	12-Jul-04	*C. horsfieldi*	*P. hypomelanus*	Pulau Tioman	Malaysia	02.84334°	104.15935°	--	--	--
7120420	2	PT20	12-Jul-04	*C. horsfieldi*	*P. hypomelanus*	Pulau Tioman	Malaysia	02.84334°	104.15935°	--	--	--
7120421	2	PT21	12-Jul-04	*C. horsfieldi*	*P. hypomelanus*	Pulau Tioman	Malaysia	02.84334°	104.15935°	1	1	1
7120422	1	PT22	12-Jul-04	*C. horsfieldi*	*P. hypomelanus*	Pulau Tioman	Malaysia	02.84334°	104.15935°	--	--	--
7120423	1	PT23	12-Jul-04	*C. horsfieldi*	*P. hypomelanus*	Pulau Tioman	Malaysia	02.84334°	104.15935°	1	1	1
7120424	2	PT24	12-Jul-04	*C. horsfieldi*	*P. hypomelanus*	Pulau Tioman	Malaysia	02.84334°	104.15935°	1	1	1
7120425	2	PT25	12-Jul-04	*C. horsfieldi*	*P. hypomelanus*	Pulau Tioman	Malaysia	02.84334°	104.15935°	1	1	1
7120426	2	PT26	12-Jul-04	*C. horsfieldi*	*P. hypomelanus*	Pulau Tioman	Malaysia	02.84334°	104.15935°	1	1	1
7120427	3	PT27	12-Jul-04	*C. horsfieldi*	*P. hypomelanus*	Pulau Tioman	Malaysia	02.84334°	104.15935°	1	1	1
7120428	2	PT28	12-Jul-04	*C. horsfieldi*	*P. hypomelanus*	Pulau Tioman	Malaysia	02.84334°	104.15935°	1	1	1
7120429	2	PT29	12-Jul-04	*C. horsfieldi*	*P. hypomelanus*	Pulau Tioman	Malaysia	02.84334°	104.15935°	1	1	1
7120433	2	PT33	12-Jul-04	*C. horsfieldi*	*P. hypomelanus*	Pulau Tioman	Malaysia	02.84334°	104.15935°	--	--	--
7120434	2	PT34	12-Jul-04	*n/a*	*P. hypomelanus*	Pulau Tioman	Malaysia	02.84334°	104.15935°	--	--	--
7120436	2	PT36	12-Jul-06	*C. horsfieldi*	*P. hypomelanus*	Pulau Tioman	Malaysia	02.84334°	104.15935°	--	--	--
Rest1	2	LYL1	4-Feb-06	*C. horsfieldi*	*P. lylei*	Phnom Penh	Cambodia	11.55815°	104.91740°	1	1	1
155642	2	LYL2	8-Jan-06	*C. horsfieldi*	*P. lylei*	Soc Trang	Vietnam	09.57849°	105.97201°	1	1	1
155657	2	CM1	11-Jan-06	*C. horsfieldi*	*P. vampyrus*	Ca Mau	Vietnam	09.15258°	104.91347°	1	1	1
155660	3	CM2	12-Jan-06	*C. horsfieldi*	*P. vampyrus*	Ca Mau	Vietnam	09.15258°	104.91347°	1	1	1
155661	1	CM3	12-Jan-06	*C. horsfieldi*	*P. vampyrus*	Ca Mau	Vietnam	09.15258°	104.91347°	1	1	1
701043	2	KB1	1-Jul-04	*C. horsfieldi*	*P. vampyrus*	Kuala Berang	Malaysia	05.07219°	103.01707°	1	1	1
702041	1	KB2	2-Jul-04	*C. horsfieldi*	*P. vampyrus*	Kuala Berang	Malaysia	05.07219°	103.01707°	1	1	1
702043	1	KB3	2-Jul-04	*n/a*	*P. vampyrus*	Kuala Berang	Malaysia	05.07219°	103.01707°	1	1	1
702044	1	KB4	2-Jul-04	*C. horsfieldi*	*P. vampyrus*	Kuala Berang	Malaysia	05.07219°	103.01707°	1	1	1
709041	2	KB5	9-Jul-04	*E. sundaicum*	*P. vampyrus*	Lenggong	Malaysia	05.13051°	100.83254°	1	1	1
RC14	2	RC14	9-Jul-04	*C. horsfieldi*	*P. vampyrus*	Lenggong	Malaysia	05.13051°	100.83254°	1	1	1
RC24	1	RC24	8-Jul-04	*C. horsfieldi*	*P. vampyrus*	Lenggong	Malaysia	05.13051°	100.83254°	1	1	1
RC29	1	RC29	8-Jul-04	*C. horsfieldi*	*P. vampyrus*	Lenggong	Malaysia	05.13051°	100.83254°	1	1	1

GenBank accession numbers: CytB (KF273687 - KF273736); CoI (KF273737 - KF273783); and CoII (KF273784 - KF273833).

Bat fly specimens examined, morphological ID, host species, collection locality name and coordinates, and genes sequenced for one randomly selected bat fly from each bat.

### Laboratory methods

DNA was extracted from one randomly selected fly per bat using a technique where 1–3 legs per fly were removed (allowing retention of fly voucher). Qiagen DNeasy tissue extraction kits were used per manufacturer’s protocol with a 24 hr tissue digestion, and two combined elutions of 50ul of Buffer AE warmed to 55°C.

Bat flies were sequenced at three mtDNA genes, cytochrome B (*cytB*), cytochrome oxidase II (*CoII*), and cytochrome oxidase I (*CoI*), that were expected to show genetic differences at the population level based on previous intraspecific studies of Diptera [36-39]. Published forward and reverse primers used to amplify each gene were as follows: for *cytB*, A5 and B 1.1 [36]; for *CoII*, A-tLEU and B-tLYS [40]; and for *CoI* LepF1 and LepR1 [41]. For the *cytB* and *CoII* genes, we used PuReTaq Ready-To-Go™ PCR Beads (GE Healthcare) with 21 μl of molecular grade ddH_2_0, 1 μl of each primer [10 mM], and 2 μl of template DNA. PCR conditions for *cytB* were identical to those in Dittmar and Whiting (2003), and for the *CoII* PCR conditions were an initial denaturation period of 3 min at 94°C, followed by 35 cycles of 94°C for 1min, 47°C for 1min, and 74 C for 1min, with a final extension at 74°C for 7min. PCR mix and cycling conditions for *CoI* followed Hebert *et al.*[41] although total reaction volume was reduced in half to 25 μl. Attempts to amplify the mtDNA control region were unsuccessful using primers and conditions from Oliveira *et al.*[42], multiple stutter bands were amplified and a single PCR product was never obtained for the control region. Negative controls were always included in PCR reactions to assess possible contamination.

PCR products were cleaned using Agencourt (Beverly, Massachusetts) AmPure magnetic beads, cycle sequenced with Big Dye v.3.0 terminator mix (Applied Biosystems, Inc., Foster City, California), and final DNA was precipitated using Agencourt CleanSeq magnetic beads. All sequencing was performed on an ABI 3730xl capillary sequencer (Applied Biosystems) at the Sackler Institute for Comparative Genomics at the American Museum of Natural History, New York. Sequences were edited in Sequencher v.4.6 and manually corrected for ambiguous base calls. Alignment of sequences was done using MAFFT v.6 using default parameters [43]. Aligned sequences were trimmed using MacClade 4.08 [44]. No insertions or deletions were present in sequences of any of the three genes.

### Phylogenetic and population genetic analyses

Phylogenetic relationships and nucleotide/haplotype diversity were initially determined for each mtDNA gene fragment independently. As there was a paucity of variable and parsimony-informative sites for each marker alone, sequence data from the three mtDNA genes were concatenated for a total of 1744 bp for each specimen. Phylogenetic analyses on the pooled dataset were conducted using PAUP* for maximum parsimony (MP) analysis [45], RAxML v.7.0 for maximum likelihood (ML) analysis [46], and Mr. Bayes for Bayesian analysis [47]. Nodal support was evaluated with the nonparametric bootstrap method [48]. MP bootstrap analysis used 1000 replicates, TBR branch swapping, with a starting tree obtained by random stepwise addition and additional sequences added at random with 10 replicates. The optimum model for ML analysis was determined using Akaike’s Information Criterion to be GTR + G using Modeltest v.3.7 [49]. Optimal evolutionary models did not differ by locus, and data were not partitioned. RAxML analysis of 1000 bootstrap runs beginning with a random seed, and user-defined outgroup, was implemented on the CIPRES Portal webserver found at http://www.phylo.org/sub_sections/portal/. Bayesian analysis was conducted using MrBayes version 3.2 [47], with data partitioned by gene. The GTR + gamma + i model of evolution was used with a flat prior for topologies, uniform priors on gamma and alpha parameters, an unconstrained exponential prior on branch lengths, and Dirichlet priors on all other parameters. A total of ten million generations were run, and convergence was assessed using AWTY [50]. Sequences obtained from *Eucampsipoda sundaica* (#KB5) were used as an outgroup to *Cyclopodia horsfieldi* in all phylogenetic analyses because of its basal position within the Cyclopodiinae [14].

Aligned FASTA files were collapsed into variable sites and haplotypes for parsimony network reconstruction with the online tool FaBox 1.31 (http://users-birc.au.dk/biopv/php/fabox/). Fifty-one bat flies were sequenced, but outgroup and individual flies that did not have complete sequences for all three genes were excluded in subsequent analyses (PER08, PER02, PT15, PT12, and KB1), leaving 45 individuals. Statistical parsimony networks, useful for inferring relationships among sequences that have recently diverged, were created using TCS v.1.21 [51]. Parsimony networks were explored using the default connection limit of 95%, but due to one divergent haplotype in the network (RC24), final network was determined with a user-defined limit set to 19 steps. Haplotype (Hd) and nucleotide (π) diversity [52] were calculated in DnaSP v.4.5 [53]. Pairwise F_ST_ values were calculated in Arlequin using a method based on pairwise differences in sequence data, and statistical significance was assessed with 1000 permutations [54]. We tested for isolation by distance between *C. horsfieldi* populations from Malaysia only, using a Mantel test [55] with 1000 permutations implemented in the R package adegenet 1.3-6 [56]. All sequences are available on GenBank: CytB (KF273687 - KF273736); CoI (KF273737 - KF273783); and CoII (KF273784 - KF273833).

Analysis of molecular variance (AMOVA), was used to test hypotheses regarding the partitioning of genetic variation for *C. horsfieldi* among host species, among sampling localities within host species, and among individuals within sampling localities [57]. AMOVA was conducted in Arlequin v.3.1 [58] using the standard haplotype format with statistical significance assessed by 1000 permutations.

## Results

### Bat fly morphology and demography

**Figure 2:**
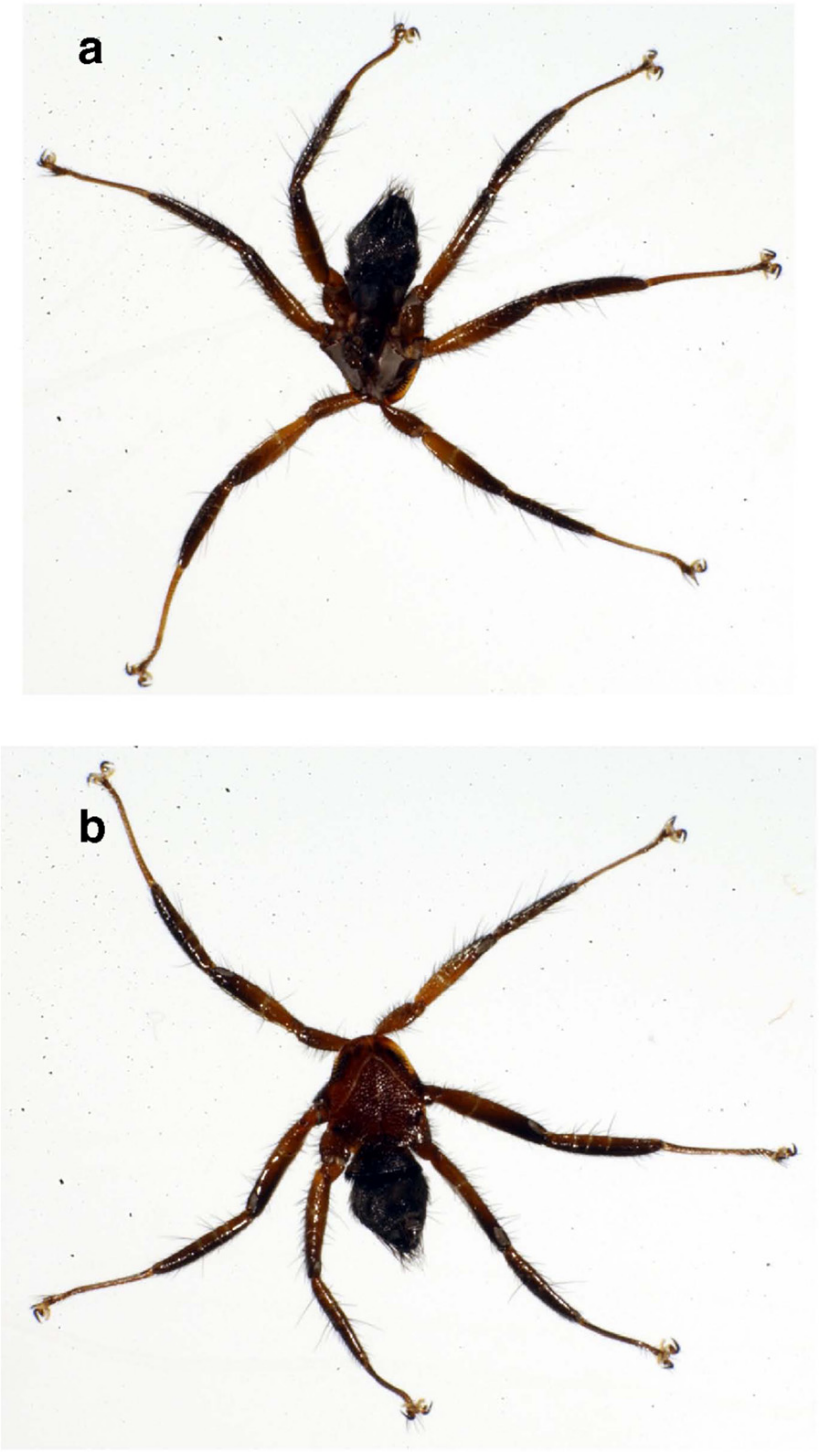
***Cyclopodia horsfieldi *collected from *Pteropus hypomelanus, *dorsal view (a) and ventral view (b)*.*** Photographs were prepared using a Microptics^TM^ ML-1000 digital imaging system.

**Figure 3:**
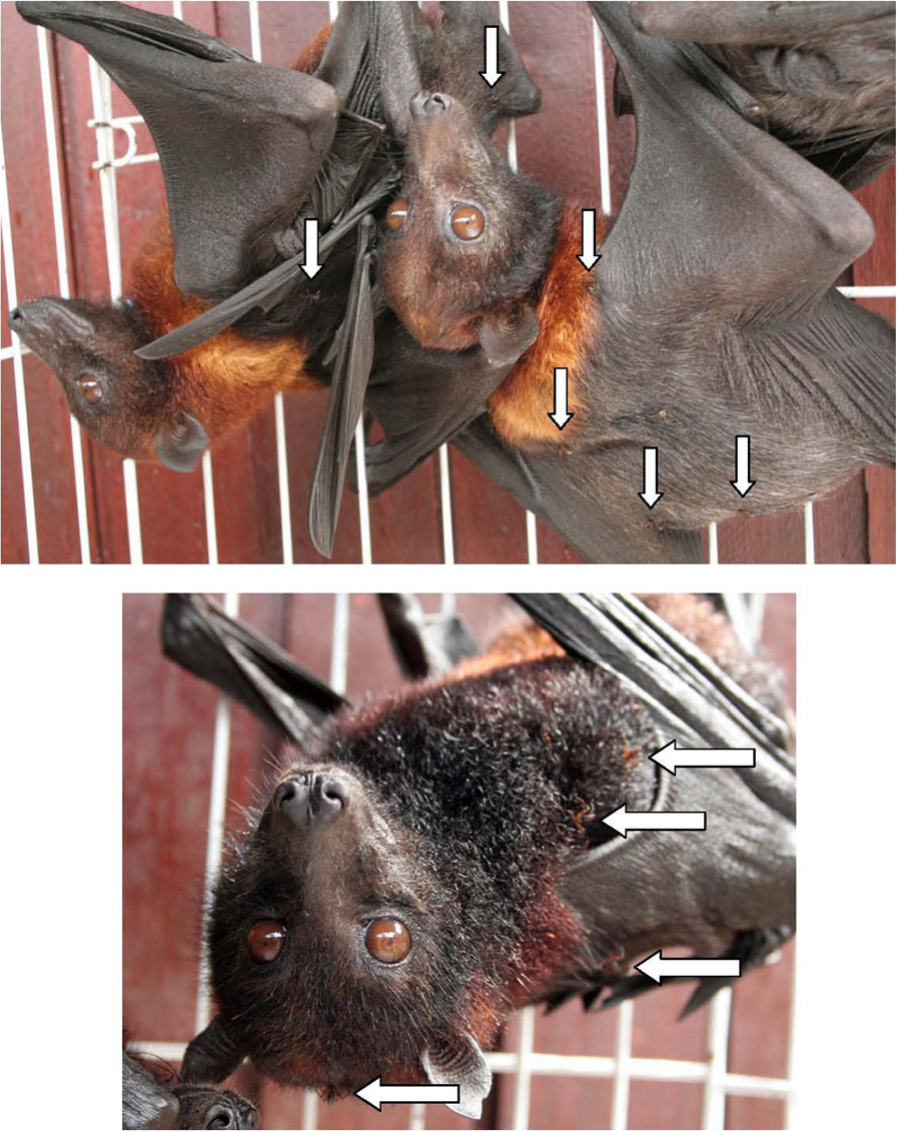
***Pteropus hypomelanus *individuals from Pulau Pangkor, Malaysia infested with *C. horsfieldi *(shown with arrows).**

All bat fly specimens examined were all identified morphologically as *Cyclopodia horsfieldi* (Figure 2) with the exception of two individuals of *Eucampsipoda sundaica*[59] collected from a *Pteropus vampyrus* (#0709041) from Kuala Berang, Malaysia (Table 1). Some intraspecific morphological variation was noted, including minor differences in counts of ctenidial spines on male sternite and counts of dorsal abdominal setae on the females, but no systematic character variation related to host species or geography was observed. The sex ratio of *C. horsfieldi* specimens examined was male-biased, 1.85♂ to 1♀. We observed that most bats were parasitized by at least one fly, and that *Pteropus hypomelanus* individuals had higher numbers of parasites than *P. vampyrus*. Several *P. vampyrus* captured hosted no flies but this was very rarely observed for *P. hypomelanus*. Many *P. hypomelanus* examined harbored 4+ flies per individual (Figure 3); and some individuals had 10+ flies. Unfortunately, bats were not exhaustively sampled for flies and the number of fly specimens collected was generally limited to a few flies per bat regardless of parasite load, and quantitative data on intensity of infestation was not collected. The number of bat flies collected from each host was not significantly different (at the p<0.05 level) between *P. vampyrus* and *P. hypomelanus* using both a standard *t*-test (t=−1.85; df=13.8; p-value=0.085) and Mann–Whitney test (W=176.5; p-value=0.066). We also inadvertently collected *Neolaelaps spinosa* (Acari: Mesostigmata) mites as we removed individual flies with forceps. These mites appear to be phoretic with bat flies [60], a relationship that has been documented with mites and other dipteran species [61-63].

### Mitochondrial sequence variation, Cyclopodia horsfieldi

Average nucleotide diversity for the combined *C. horsfieldi* mtDNA dataset (3 gene fragments, 1744 bp) was 0.002 ± .0004 (Table 2); comparable to values found in other Dipteran species [64-66]. The gene fragment length (base pairs, bp) and variable/parsimony informative sites for each gene segment were as follows: *CoI* (702bp, 11/5), *CoII* (678bp, 11/2), and *cytB* (364bp, 8/1). There was an overall high amount of haplotype diversity across all markers and specimens (0.903 ± .026). Although sample sizes were limited, overall nucleotide and haplotype diversity were both higher for *C. horsfieldi* individuals from *Pteropus vampyrus* (π=0.0042±0.0017, hd=0.972±0.064) compared to *P. hypomelanus* (π=0.0014±0.00009, hd=0.861±0.042) (Table 2). *Cyclopodia horsfieldi* individuals from *Pteropus hypomelanus* on Pulau Pangkor had the lowest haplotype diversity of any population (0.417±0.191), which corresponded with the smallest host population census size (n=200 bats). Total A-T content was uniformly high for all sequences (77.3%), as is common in other insect mitochondrial genomes [67,68]. Summary of base frequencies across the complete dataset of 51 sequenced fly individuals were: A=0.3546, C=0.1196, G=0.1006, and T=0.4251.

**Table 2:** **Nucleotide and haplotype diversity for *Cyclopodia horsfieldi *by host species and locality**

**Host spp./locality**	**# of Sequences**	**# of Haplotypes**	**Haplotype diversity ± SD**	**# Variable sites**	**Nucleotide diversity ± SD**
*P. vampyrus* (Malaysia)	6	6	1.0 ± 0.096	23	0.0053 ± 0.002
*P. vampyrus* (Vietnam)	3	3	1.0 ± 0.272	6	0.0023 ± 0.001
*P. vampyrus* (ALL)	9	8	0.972 ± 0.064	25	0.0042 ± 0.001
*P. hypomelanus* (PT)	14	8	0.912 ± 0.049	7	0.0012 ± 0.000
*P. hypomelanus* (PPH)	9	3	0.417 ± 0.191	2	0.0003 ± 0.000
*P. hypomelanus* (PER)	10	6	0.867 ± 0.085	8	0.0019 ± 0.000
*P. hypomelanus* (ALL)	34	12	0.861 ± 0.042	11	0.0014 ± 0.000
*P. lylei* (PP and ST)	2	2	1.000 ± 0.500	4	0.0023 ± 0.001
TOTAL	45	17	0.903 ± 0.026	29	0.0020 ± 0.000

Data shown only for individuals (n=45) with complete combined mtDNA dataset of CoI, CoII, cytB (1744 bp).

### Population genetic structure, Cyclopodia horsfieldi

**Figure 4:**
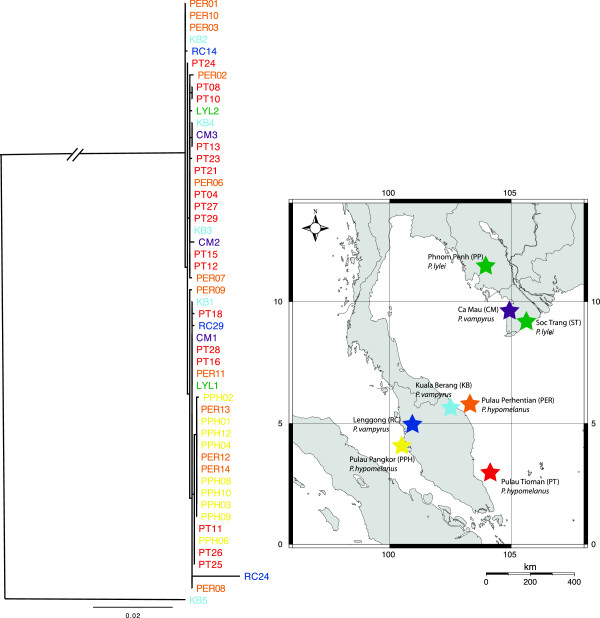
**Maximum likelihood phylogeny with sampling map, *Cyclopodia horsfieldi.***

**Figure 5:**
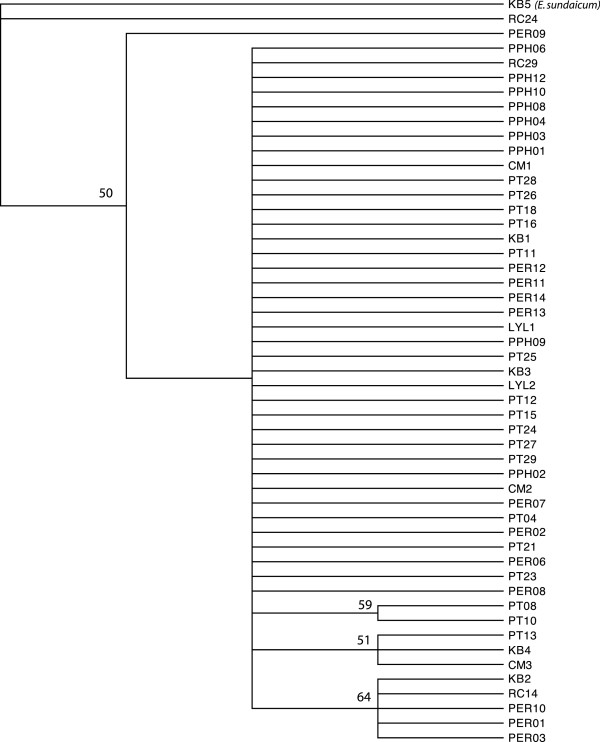
**Maximum parsimony phylogeny, majority-rule consensus tree, *Cyclopodia horsfieldi.***

**Figure 6:**
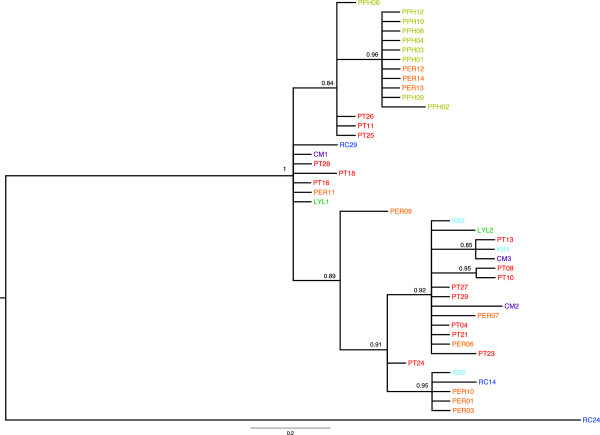
**Bayesian phylogeny *Cyclopodia horsfieldi *using concatenated dataset, GTR + gamma + i model of evolution partitioned by gene, ten million generations, and no outgroup*.***

**Figure 7:**
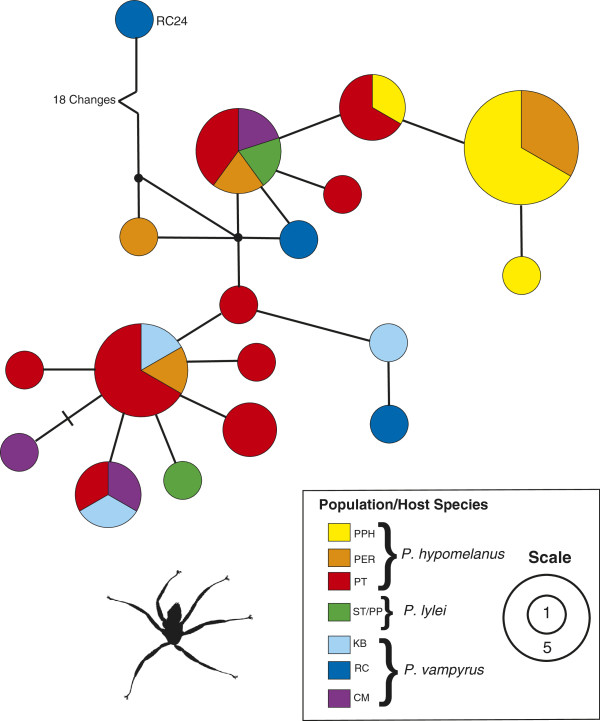
**Statistical parsimony network, combined mtDNA dataset, *Cyclopodia horsfieldi.***

Phylogenetic analyses of the combined mtDNA data set using ML (Figure 4) and MP (Figure 5) showed little resolution of population genetic structure for *C. horsfieldi* sampled across sites in Southeast Asia. No significant population structure that corresponded to either geography or host species was detected in any of the analyses (Figures 4, 6, 7). Despite an almost complete lack of resolution, some flies from geographically distant areas formed clades with greater than 50% support in the MP tree (e.g. PT13, KB4, and CM3, from Peninsular Malaysia and Vietnam), also evident in the Bayesian tree (Figure 6). Bat flies collected from Pualu Pangkor (PPH), off the west coast of Peninsular Malaysia, appear to be distinct in the haplotype network (Figure 7, yellow), but several individuals shared identical haplotype sequences with flies collected 300 km away on the east coast of Malaysia from Pulau Perhentian (PER) (Figure 7). The most genetically divergent bat fly individual, RC24, was a male morphologically indistinguishable from all the other *Cyclopodia horsfieldi* specimens.

Despite an overall lack of geographic structure observed in phylogenetic and haplotype network analyses, pairwise F_ST_ values for fly populations from Peninsular Malaysia fit *a priori* predictions based on gene flow of their *Pteropus* hosts [69]. Significant pairwise F_ST_ values were observed between *Cyclopodia horsfieldi* sampled from *Pteropus hypomelanus* from western (PPH) vs. eastern (PER, PT) island populations (Table 3), suggesting some limits to parasite gene flow between these islands. Similarly, average pairwise F_ST_ between *P. vampyrus* and the western *P. hypomelanus* population (PPH) was significant and high (F_ST_ =0.443) (Table 3). In contrast, pairwise F_ST_ values between flies collected from the two western island *P. hypomelanus* populations were low and non-significant, as were F_ST_ values between bat flies from *P. vampyrus* vs. eastern *P. hypomelanus* populations (PER, PT) (Table 3). The mantel test for geographic isolation by distance was rejected (p=0.373). The relationship between *Pteropus hypomelanus* F_ST_ values and F_ST_ from *Cyclopodia horsfieldi* flies collected from the same bat populations was strong (r=0.971) but non-significant in a randomized mantel test (p=0.34).

**Table 3:** **Pairwise F**_**ST **_**values (below diagonal) and geographic distances in kilometers (above diagonal) for *Cyclopodia horsfieldi *populations from *Pteropus vampyrus *and three *P. hypomelanus *populations**

	***P. vampyrus***^**1**^	**PPH**^**2**^	**PT**^**2**^	**PER**^**2**^
***P. vampyrus***	--	170	375	135
**PPH**	0.443*	--	430	305
**PT**	0.027	0.560*	--	375
**PER**	0.027	0.435*	0.031	--

Data used for Isolation by Distance mantel test.

* Significant based on 1000 permutations; p<0.05. Pairwise F_ST_ calculated with complete mtDNA dataset (1744 bp).

^1^*P. vampyrus* from Peninsular Malaysia only, RC and KB pooled.

^2^*P. hypomelanus* from Pulau Pangkor, PPH; Pulau Tioman, PT; and Pulau Perhentian, PER.

AMOVA results support a lack of geographic and host-specific population genetic structure in *Cyclopodia horsfieldi* (Table 4). Molecular variance was primarily partitioned within populations (77.3%), not between geographic localities within host species (22.3%) or between host species (0.4%, not significant).

**Table 4:** **Hierarchical analysis of molecular variance (AMOVA) for combined mtDNA dataset (1744bp), *Cyclopodia horsfieldi***

**Source of variation**	**d.f.**	**S.S.**	**Variance**	**Variation %**	**Fixation indices**	***p *value**
Among host species	2	7.51	0.008	0.4	Φ_CT_ = 0.0038	0.445
Among populations within host species	3	16.92	0.47	22.3	Φ_SC_ = 0.224	<0.002
Within populations	38	61.87	1.63	77.3	Φ_ST_ = 0.227	<0.001

Populations grouped within host species, *Pteropus hypomelanus* (PPH, PT, PER); *P. vampyrus* (Malaysia, Vietnam); and *P. lylei* (two individuals, considered one population).

## Discussion

### Host specificity

Morphological and molecular species identification revealed that one fly species, *Cyclopodia horsfieldi*, parasitized all three *Pteropus* host species sampled from Malaysia, Vietnam and Cambodia. The only exceptions were two individuals of *Eucampsipoda sundaica* collected from an individual *Pteropus vampyrus* in Peninsular Malaysia. Minor morphological differences were observed among individuals of *Cyclopodia horsfieldi*, but these did not correlate with sampling locality or host species, and the lack of systematic morphological variation is supported by a similar lack of geographic or host-specific population genetic structure. The sex ratio of the bat flies we randomly sampled was male-biased. This observation agrees with previous reports of excess males in bat fly populations, and may be caused by grooming mortality patterns [70] or the diurnal activity of female flies during larviposition [71].

Experimental studies have shown that some bat flies exhibit behavioral preference towards their primary host over congeneric and confamilial species [72]. However, we observe a natural case of parasite oligoxeny among three bat species in Southeast Asia, which has also been documented in *Cyclopodia spp.* from flying foxes in Australia [16]. Oligoxeny is more likely in hosts that share geographic and habitat niches and/or co-roost at the same sites, and it appears to be an evolutionary advantageous strategy for an ectoparasite [2]. Our results suggest that co-mingling of *Pteropus* spp. in Southeast Asia is more common than previously assumed. On occasion, *Pteropus vampyrus/P.lylei* and *P. vampyrus/P. hypomelanus* co-roosting has been observed in the same trees in Ca Mau, Vietnam and Pulau Langkawi, Malaysia, respectively [69]; and similar observations have been made in Thailand (P. Duengkae, per. comm.). It is also possible that these species could be sharing bat fly parasites without simultaneous occupation of the same roost, i.e. sequential use of a roosting site within a 2–3 week window where flies may emerge from metamorphosis on the roost substrate.

The presence of *Eucampsipoda sundaica* flies found on a *Pteropus vampyrus* individual may represent a case of host-switching, which would suggest contact and ecological overlap between other fruit bat species in Peninsular Malaysia. *Eucampsipoda sundaica* is the sole ectoparasite of the Dawn bat, *Eonycteris spelaea*[24], although there are previous records of this species from *Cynopterus sphinx* in India, *Pteropus* in Myanmar, and *Rousettus amplexicatus* from the Philippines [59,73]. Also, we found one genetically divergent bat fly, RC24, to be an outlier in haplotype network and phylogenetic analyses. This fly was morphologically indistinguishable from the other *Cyclopodia horsfieldi* examined, but could possibly represent a male of the sister species, *C. sykesii*[16]. *C. sykesii* is primarily associated with *Pteropus giganteus* from South Asia [16], and thus could possibly represent a case of host switching by secondary contact between these flying fox species. More fly specimens from *P. vampyrus* should be sampled and examined to confirm the rarity of these results.

### Comparative host-parasite population structure

Overall, we found that populations of *Cyclopodia horsfieldi* lacked population genetic structure across geographically distant sites and host species in Southeast Asia. However, pairwise F_ST_ values between some fly populations, particularly from *Pteropus hypomelanus*, corroborate expectations of reduced gene flow based on the population genetics of their flying fox hosts [69]. Olival [69] found that island populations of *P. hypomelanus* were significantly differentiated at mtDNA markers from East to West in Malaysia (e.g. F_ST_ = 0.95, mtDNA control region), but had much higher levels of gene flow between the east coast islands (F_ST_ =0.0 to 0.4). Here we observe a similar pattern for the parasite, *Cyclopodia horsfieldi,* with significant pairwise F_ST_ values between flies from Pulau Pangkor off the west coast and Pulau Tioman (F_ST_ =0.560) and Pulau Perhentian (F_ST_ =0.435) off the east coast of Malaysia. In contrast, F_ST_ values among flies from the two east coast islands were low and not significant (F_ST_ =0.031). We also observed low and non-significant F_ST_ values between mainland populations of *Pteropus vampyrus* and island populations of *P. hypomelanus*, with the exception of Pulau Pangkor (PPH). These data suggest ongoing or recent gene flow among *Cyclopodia horsfieldi* parasites between mainland *Pteropus vampyrus* and eastern *P. hypomelanus*; and less contact between *P. vampyrus* and the small western island of Pulau Pangkor.

In contrast, *Pteropus vampyrus* was found to be essentially panmictic across a very large geographic range of thousands of kilometers in Southeast Asia at mtDNA markers [69], and these data were corroborated by satellite telemetry showing regular long-distance dispersal (100 s of kilometers), lack of roost fidelity, and large home range sizes (128,000 km^2^) across Malaysia, Thailand, and Indonesia [74]. The shallow branching pattern in our ML phylogeny and mixing of haplotypes observed in the statistical parsimony network suggest that *Cyclopodia horsfieldi* populations have recently diverged or are subject to ongoing gene flow. Frequent contact between flying fox host species and subsequent ectoparasite gene flow may best explain the lack of parasite population structure observed. In particular, we suggest that the highly volant *Pteropus vampyrus* may be acting as a “vector” spreading bat flies to other conspecific populations and *Pteropus* species in the region during long-distance dispersal events. Satellite telemetry studies have shown that *P. vampyrus* uses small islands as stopover sites when migrating to and from Peninsular Malaysia and Sumatra [74]. We also observed higher nucleotide and haplotype diversity values for flies from *P. vampyrus* relative to those from *P. hypomelanus*. This suggests that populations of *Cyclopodia horsfieldi* from *Pteropus vampyrus* may have larger effective population sizes and may be acting as a source for founding island populations of parasites [12,75]. In summary, our observations of parasite population structure, combined with prior results from host population genetics and satellite telemetry, lend support to the idea that *Pteropus vampyrus* is actively dispersing parasites to the outlying island populations of *P. hypomelanus* and making contact, and not vice versa.

Three alternative explanations, beyond host-mediated gene flow of *Cyclopodia horsfieldi*, may explain our results. First, the observed lack of geographic or host species population genetic structure seen in *C. horsfieldi* could simply be due to invariability or insufficient variance of the molecular markers examined. We suggest this is not the case as a number of previous studies of winged, free-living dipterans using the same markers at similar geographic scales have found significant geographic population structure [36-39,76]. All else being equal, free-living, volant flies should have lower levels of population genetic subdivision than parasites almost wholly dependent on their hosts for dispersal (i.e. bat flies). Second, endosymbionts, e.g. *Wolbachia* or *Arsenophonus*, may have influenced the observed lack of population structure and genetic variation via mitochondrial sweeps, as seen in other dipteran species [34,39,77,78]. Differences in *Wolbachia* infection and immunity can create striking differences in mtDNA diversity and lead to speciation, e.g. in fig wasps [79]. A number of novel endosymbiont lineages have been identified in bat flies [80-82], and previous studies have suggested that selective sweeps caused by these endosymbionts may explain a lack of mtDNA diversity in bat flies [34]. While we cannot rule out this possibility, it seems unlikely that this selective pressure has influenced the demographic history of each bat fly population sampled. Also, even in the case of *Wolbachia* infection in insects, high levels of migration may still be a more prominent factor reducing genetic differentiation in species with potential to disperse long distances [83]. Third, demographic factors, i.e. populations bottlenecks with subsequent expansion, could also explain a lack of genetic variation and phylogeographic structure in *Cyclopodia horsfieldi*[84]. This scenario also seems unlikely, as it also would have to occur independently across multiple geographic localities for each sampled population. The association between *Cyclopodia spp.* and their flying fox hosts is likely not recent [16], and we believe that high gene flow among parasites is the most parsimonious explanation for the observed results.

#### Implications for zoonotic disease ecology

Bats are important reservoir hosts for a large number of emerging zoonotic viruses [85], including neurotropic viruses with high mortality rates in the genus Henipavirus [86,87]. Nipah virus (NiV) caused significant human mortality (~40%) during its initial outbreak in Malaysia in 1998 [88], and has emerged repeatedly in Bangladesh and India since 2001 [89-91]. The bat species examined here, *Pteropus vampyrus*, *P. lylei*, and *P. hypomelanus* are considered three of the most important natural reservoir hosts for this virus [92-94]. The low levels of population differentiation observed in *Cyclopodia horsfieldi*, suggest high levels of contact among *Pteropus* species in Southeast Asia – a pattern not apparent from host genealogy or prior ecological studies alone.

The data we present here on host specificity also contributes to a better understanding of interspecific contact between *Pteropus* species in Southeast Asia, and also potentially with other fruit bat species not sampled here. Two individuals of the fly *Eucampsipoda sundaica* were collected from *Pteropus vampyrus* in Kuala Berang, Malaysia. This is an ectoparasite species most commonly associated with *Eonycteris spelaea*, a cave-dwelling, nectivorous bat species in Malaysia [24], but also known from fruit bats in the genera *Cynopterus* and *Rousettus*[59,73]. This suggests that physical contact between *Pteropus spp.* and other Pteropodid species with different roosting ecologies may occur on occasion, potentially during interactions over shared food resources [95]. For example, in Bangladesh physical contact between *Pteropus giganteus*, *Rousettus leschenaultii,* and *Cynopterus brachyiotis* was observed with infrared cameras at shared date palm sap feeding sites [96]. This supports observations from serological studies of NiV in Malaysia in which neutralizing antibodies to NiV were detected in the sera of 5% (2/38) of *Eonycteris spelaea*[97]. Similarly, 3.5% (2/56) of *Cynopterus brachyotis* individuals were also positive for NiV antibodies in these surveys [97]. These results were presumed to represent rare instances of cross-species NiV spillover from *Pteropus spp.*, and fit with our observations of occasional ectoparasite sharing between these species.

We also observed one genetically divergent fly, RC24, that could represent a morphologically indistinguishable male of the sister species to *Cyclopodia horsefeldi*, *C. ferrarii*. More specimens of bat flies, especially females, collected from *Pteropus vampyrus* should be examined in the future to see if this truly represents *Cyclopodia ferrarii* and another case of parasite host switching between bat species – this time between *Pteropus vampyrus* and *P. giganteus*. Horizontal transfer of bat flies between sympatric fruit bat species deserves further research attention, as results are relevant for understanding the mechanism of cross-species viral spillover and maintenance of diseases amongst reservoir host species. NiV has a short infectious period and long-term immunity in bats, suggesting that very large populations, or metapopulation dynamics, are necessary to sustain the virus in bat reservoirs [98-100]. Host species contact inferred here using ectoparasite population genetic structure may provide insight as to how NiV can be maintained in some bat populations with relatively small census sizes, e.g. *Pteropus hypomelanus*[101], and why it may be so widespread among *Pteropus* spp in the region [92-94,102].

#### Bat flies as potential vectors for bat pathogens

Other diseases endemic in bat populations, such as apicomplexan parasites, may not cause significant morbidity or mortality to their hosts, but have evolved long-term co-evolutionary relationships with them [103]. Malaria parasites (*Hepatocystis* sp.) were identified using morphological and molecular methods from the same population of *Pteropus hypomelanus* sampled here [104]. The role of bat flies in the potential transmission of bat malarial parasites is not clear. Earlier studies failed to find evidence for *Hepatocystis* infection in bat flies [105-107], and only *Culicoides* sp. midges have been demonstrated to be competent vectors for these parasites [108]. More recently, oocysts of the parasite, *Plasmodium murinus*, were found in 7 of 26 dissected *Nycteribia kolenatii* from *Myotis daubentoni* bats [109]. Electron microscopy of the salivary glands of these flies confirmed the presence of sporozoites [109] which suggests possible transmission of malarial parasites by nycteribiids. If bat flies are suitable vectors for *Hepatocystis* and other related parasites, and if they move regularly between fruit bat populations and species (as demonstrated here) this could disrupt expected co-evolutionary patterns between chiropteran hosts and their malarial parasites.

Bat flies also play an important role in the evolution and transmission of *Bartonella* spp. in bats globally [110]. There is some evidence for long-term coevolutionary patterns between bat flies and their *Bartonella* parasites [110]. For example, *gltA* genotypes of *Bartonella* from *Cyclopodia greefi* flies collected from *Eidolon helvum* (Family Pteropodidae) in Ghana grouped closely with *Bartonella* genotypes from related *Cyclopodia horsfieldi* flies sampled from *Pteropus hypomelanus* in Malaysia, suggesting an underlying co-phylogenetic pattern for *Bartonella*-bat-bat fly associations [110]. Additional studies on bat fly population structure, dispersal, ecology and host specificity will help to clarify the role of bat hosts vs. bat ectoparasites/vectors in the evolution and ecology of *Bartonella*[110,111].

## Conclusions

For the first time, we investigate the population genetic structure of an Old World bat fly species, *Cyclopodia horsfieldi,* and show it to be a useful tool to understand host movement and interspecific contact among bat species. We observed an overall lack of morphological variation and phylogenetic structure across geographic regions and host species for *C. horsfieldi*. For some bat fly populations, elevated pairwise genetic differentiation (F_ST_) did correspond to a lack of gene flow in host populations, i.e. insular populations of *P. hypomelanus* in Malaysia. By combining our data with previous studies of bat genetics, telemetry, and parasite host range, we suggest that *P. vampyrus* may facilitate movement of bat flies through frequent physical contact among the three *Pteropus* species in the region, and occasionally with other fruit bat species. Our approach and findings have wide implications for understanding zoonotic disease dynamics and cross-species transmission in bats, in particular the transmission and ecology of Nipah virus. Bat flies may also play a critical role in bat disease transmission and evolution (e.g. *Bartonella* or apicomplexan parasites), and their ecology, dispersal, and competence as vectors of bacterial and/or viral pathogens are in need of further investigation.

## Competing interests

The authors declare that they have no competing interests.

## Authors’ contributions

KJO lead and implemented the sample collection, laboratory work, molecular analysis, and writing of the manuscript. CWD examined the morphology for all specimens and helped to draft the manuscript. KD examined specimens and helped to revise the manuscript. JCM, DM, NBS, SLP, PD, and RD assisted in the study design, project coordination, analysis, and manuscript revisions; NBS additionally helped collect specimens on Pulau Perhentian, Malaysia. All authors read and approved the final manuscript.

## Authors’ information

Dr. Olival is currently a Senior Research Scientist at EcoHealth Alliance. His research focuses on understanding the drivers of zoonotic disease emergence and the ecology and evolution of bats, their associated parasites and viruses. This study was part of his dissertation research at Columbia University with the laboratory work completed at the Sackler Institute for Comparative Genomics at the American Museum of Natural History.
